# Comparison of the efficacy of cefoperazone-sulbactam and other cephalosporins in the treatment of infections: A systematic review and meta-analysis

**DOI:** 10.1097/MD.0000000000042182

**Published:** 2025-04-25

**Authors:** Wenwei Sun, Jieying Zhang, Ke Miao, Li Ju, Hengjie Yuan

**Affiliations:** a Tianjin Medical University General Hospital, Tianjin, China; b First Teaching Hospital of Tianjin University of Traditional Chinese Medicine, Tianjin, China.

**Keywords:** cefoperazone-sulbactam, cephalosporins, efficacy, infections, meta-analysis, multidrug-resistant organisms, safety

## Abstract

**Background:**

Cefoperazone-sulbactam is a broad-spectrum antibiotic known for its activity against a wide range of pathogens, including multidrug-resistant organisms (MDROs). This systematic review and meta-analysis aim to compare the efficacy and safety of cefoperazone-sulbactam with other cephalosporins in the treatment of infections.

**Methods:**

We conducted a comprehensive search of PubMed, Web of Science, Scopus, Cochrane Library, Medline, EMBASE, and CNKI databases for relevant studies up until July 11, 2024. Randomized controlled trials (RCTs) that compared cefoperazone-sulbactam with other cephalosporins in treating infections were included. Data were analyzed using RevMan 5.3 software, and the relative risk (RR) and mean difference (MD) were calculated. Sensitivity analysis and subgroup analyses were performed to ensure result robustness.

**Results:**

Seven RCTs involving 1017 patients were included in the analysis. Cefoperazone-sulbactam demonstrated a significantly higher treatment success rate (RR = 1.08, 95% CI [1.02–1.13], *P* = .003) and superior microbial clearance rate (RR = 1.22, 95% CI [1.11–1.34]) compared to other cephalosporins. Sensitivity analyses confirmed the stability of these findings. Adverse reactions were similar between groups, with cefoperazone-sulbactam demonstrating good safety and tolerability.

**Conclusion:**

Cefoperazone-sulbactam shows superior efficacy compared to other cephalosporins in the treatment of infections, particularly in cases involving multidrug-resistant organisms. It also exhibits a comparable safety profile, making it a valuable option in clinical practice. However, further multicenter RCTs are needed to fully assess its potential in broader clinical applications.

## 1. Introduction

Intra-abdominal infections (IAIs) represent a broad spectrum of diseases, ranging from simple acute appendicitis to severe conditions such as complex diffuse peritonitis. These infections can be triggered by various causes, including gastrointestinal perforation, appendicitis, cholecystitis, and postoperative complications.^[1]^ The severity of IAIs varies depending on the extent and complexity of the infection, typically classified into complicated intra-abdominal infections (CIAIs) and uncomplicated intra-abdominal infections (NCIAIs). CIAIs often involve organ dysfunction or the spread of localized infection throughout the abdominal cavity, leading to higher mortality rates and increased treatment complexity.^[2]^ According to the CIAO study (the Global Complicated Intra-Abdominal Infection Observational Study, CIAOW) and the World Society of Emergency Surgery (WSES) Complex Intra-Abdominal Infection Score study, the mortality rates of IAIs are 7.5%, 10.5%, and 9.2%, respectively. These data suggest that IAIs remain a significant global challenge in clinical management.^[3,4]^ Epidemiological surveys have also shown regional differences in the incidence of IAIs, with higher mortality rates in developing countries due to limited healthcare resources for managing CIAIs.^[5,6]^

The key to managing IAIs lies in effective source control and appropriate antibiotic therapy. Among all treatment strategies, source control is widely regarded as crucial because it not only prevents further spread of infection but also reduces ongoing microbial contamination, significantly improving patient outcomes.^[7]^ Methods of source control include surgical interventions (such as the resection of infected organs or drainage of abscesses) and noninterventions like percutaneous drainage. Although there are no RCTs explicitly assessing the efficacy of these interventions, existing data indicate that inadequate source control significantly increases mortality and poor prognosis, further highlighting its central role in the treatment of IAIs. Alongside source control, antibiotic therapy is another essential component in managing IAIs^[8,9]^ subsequent bacterial contamination. In certain uncomplicated cases of IAIs (such as some instances of acute appendicitis and acute cholecystitis), antibiotic therapy alone has been shown to be an effective treatment without the need for surgical intervention. However, in complicated IAIs, antibiotic selection is influenced by resistant bacterial strains, particularly ESBL-producing Enterobacteriaceae, which are more commonly associated with hospital-acquired infections.^[10]^ ESBL-producing strains can hydrolyze and inactivate a wide range of β-lactam antibiotics, including third-generation cephalosporins, penicillins, and aztreonam, significantly complicating treatment.^[11,12]^

Although third-generation cephalosporins (such as cefotaxime or ceftriaxone) remain the empirical treatment of choice for uncomplicated community-acquired IAIs, antibiotic selection in complicated IAIs must be more cautious, particularly when addressing multidrug resistant strains.^[13,14]^ In recent years, cefoperazone-sulbactam, a broad-spectrum antibiotic, has been widely used to treat various acute bacterial infections. Notably, cefoperazone-sulbactam has demonstrated strong in vitro antibacterial activity against ESBL-producing Enterobacteriaceae and carbapenem-resistant Acinetobacter baumannii, without being affected by the inoculum effect.^[15,16]^

However, despite the potential clinical utility of cefoperazone-sulbactam, there is currently no systematic meta-analysis comparing its efficacy and safety with other commonly used cephalosporins in the treatment of IAIs. Therefore, we conducted a comprehensive meta-analysis to provide high-quality evidence on the use of cefoperazone-sulbactam in treating IAIs. By summarizing the available clinical data, our study aims to offer more robust guidance for antibiotic selection in clinical practice, especially in managing CIAIs.

## 2. Methods

### 2.1. Search strategy

This systematic review and meta-analysis were conducted according to the Preferred Reporting Items for Systematic Reviews and Meta-Analyses (PRISMA) guidelines. We searched the databases of PubMed, Web of Science, Scopus, Cochrane Library, Medline, EMBASE, and CNKI for articles published from their inception until July 11, 2024. The search strategy was developed using a combination of Medical Subject Headings (MeSH) and free text terms. We searched for terms related to “Cephalosporins,” “cefoperazone/sulbactam,” and “infections” both as subject headings and free text terms, and the search results were combined to obtain the final result. For example, our search string in PubMed was: (((((Cephalosporins[MeSH Terms]) OR (cephalosporin[Title/Abstract])) OR (cephalosporins[Title/Abstract])) OR (cephalosporin antibiotics[Title/Abstract])) AND (((((cefoperazone/sulbactam[MeSH Terms]) OR (cefoperazone-sulbactam[Title/Abstract])) OR (sulperazone[Title/Abstract])) OR (sulbactam[Title/Abstract])) OR (cefoperazone[Title/Abstract]))) AND ((((infections[MeSH Terms]) OR (Intra-abdominal infections[Title/Abstract])) OR (peritonitis[Title/Abstract])) OR (abdominal sepsis[Title/Abstract])). Additionally, to ensure comprehensive and accurate results, we manually searched the reference lists of relevant studies.

### 2.2. Inclusion and exclusion criteria

The inclusion criteria for this study were: (1) randomized controlled trials (RCTs), with no restrictions on publication year or language; (2) studies focusing explicitly on IAIs; (3) studies including primary or postoperative secondary infections diagnosed by physicians using clear diagnostic criteria; (4) studies reporting cefoperazone/sulbactam as an intervention and documenting the clinical response of patients to cefoperazone/sulbactam treatment, with a control group treated with other cephalosporins and the clinical response documented.

The exclusion criteria were: (1) materials classified as reviews, letters, conference reports, or protocols; (2) IAIs combined with other types of non-abdominal infections; (3) studies not reporting the location or time of investigation; (4) duplicate publications; (5) studies with unavailable data or those that could not be accessed through other methods; (6) studies where the full text could not be obtained.

### 2.3. Study selection and data extraction

The titles and abstracts of all studies identified through the search were imported into the Endnote20 reference management software. Next, 2 authors (A and B) independently screened the studies based on their titles and abstracts. The eligibility of all relevant full-text articles was assessed according to the inclusion criteria. Two authors independently completed the eligibility assessment. Discrepancies were resolved through consensus with authors C and D. For selected studies, 2 reviewers read the full texts and independently extracted data using predesigned forms. The extracted data included: (1) basic study characteristics: first author, publication year, study location, and sample size; (2) participant details such as age, sex, body weight, education level, and family status; (3) drugs used in the intervention and control groups; (4) outcomes: the primary outcome was the clinical efficacy of cefoperazone/sulbactam, while secondary outcomes included microbial clearance rates, significant clinical efficacy rates, and adverse reactions. The extracted data were cross-checked by the 2 reviewers. Any differences were resolved through team discussions.

### 2.4. Quality assessment of the studies

The risk of bias in the collected literature was assessed using the procedures outlined in the Cochrane Handbook for Systematic Reviews of Interventions, version 5.1.0. The assessed domains included the randomization process, allocation concealment, blinding methods, reporting of study results, the presence of other sources of bias, and selective reporting of outcomes. The findings were categorized as follows: “yes” indicated accurate methods or complete data, suggesting a low risk of bias; “unclear” indicated a moderate risk of bias; “no” indicated incorrect methods or incomplete data, suggesting a high risk of bias. The data were imported into RevMan 5.4 software, which was used to generate a risk of bias assessment graph.

### 2.5. Statistical analysis

Data analysis was conducted using the RevMan 5.3 software. In the meta-analysis, we estimated the combined relative risk (RR) for categorical variables and the mean difference (MD) for continuous variables. The level of heterogeneity was assessed using the I² index, where values below 30% indicated low heterogeneity, values between 31% and 50% indicated moderate heterogeneity, and values above 50% indicated substantial heterogeneity. If significant heterogeneity was observed (I² > 50%), a random-effects model was applied. Otherwise, a fixed-effects model was used. Additionally, to examine the comparison results for different cephalosporins used in the control groups, we conducted a subgroup analysis based on the type of cephalosporin. The calculated outcome measures and their corresponding 95% confidence intervals (CIs) were illustrated in forest plots. To determine statistical significance, a *P*-value of <.05 was considered indicative. Finally, a sensitivity analysis was conducted by sequentially excluding studies to assess the stability of the results.

## 3. Results

### 3.1. Search results

**Figure 1.**
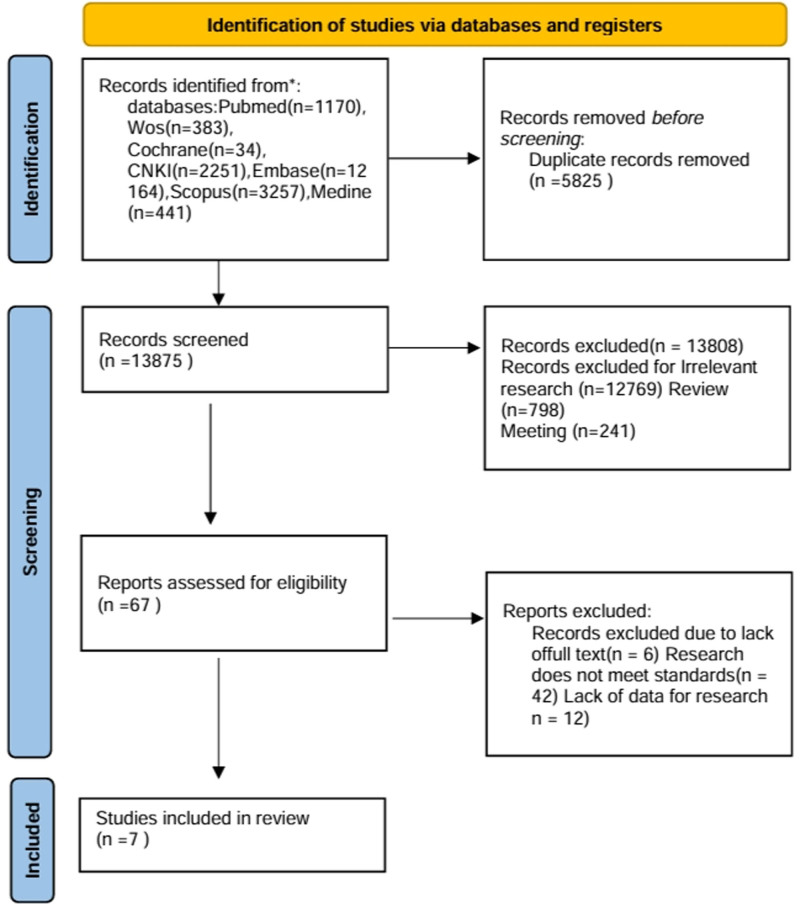
Flow diagram showing the screening and selection process of reports to be included in the meta-analysis.

Initially, we identified 19,700 potential studies using the specified search terms. Of these, 5825 duplicate studies were excluded using EndNote 20. After reviewing the titles and abstracts, 12,769 studies were deemed irrelevant and excluded. Additionally, 1039 citations were discarded as they were reviews or conference materials. Subsequently, 67 full-text articles were thoroughly reviewed. Of these, 42 articles were excluded for reasons such as nonconformity between the treatment and control groups, retrospective study design, or unavailability of the full text or study data. Ultimately, after careful examination, 7 clinical studies met the criteria and were deemed suitable for inclusion in the meta-analysis.^[17–23]^ Figure 1 illustrates the selection process.

### 3.2. Characteristics of included studies

The seven included trials involved a total of 1017 patients, with 513 in the treatment group and 504 in the control group.^[17–23]^ Among these studies, 3 were multicenter trials,^[17,22,23]^ with one study conducted across 17 different medical institutions in India,^[17]^ one in Japan,^[22]^ and one in China.^[23]^ The remaining studies were single-center trials. The control groups used different types of cephalosporins: 2 studies used ceftazidime,^[17,21]^ 3 used ceftriaxone,^[19,20,23]^ and 2 used cefotaxime.^[18,22]^ The clinical response to cefoperazone/sulbactam was assessed according to guidelines for clinical trials on antibacterial agents. A significant clinical response was defined as follows: if the infection was treated with the designated study antibiotics alone, with no recurrence, and no other antibiotics were used during the follow-up period. A clinical response was considered effective if the primary infection was controlled without the need for additional antibiotics to manage intra-abdominal infection, but the patient exhibited ongoing clinical impairment or recurrent infection. Microbial eradication was defined as the elimination of the infectious source, with no residual organisms in cultures or no growth in cultures from remaining drainage fluid. Table 1 provides details of the characteristics of the included studies, such as sample size for treatment and control groups, age of patients in both groups, and the treatment regimens used. In terms of efficacy outcomes, 7 studies assessed the overall treatment efficacy, while 5 studies^[18–22]^ evaluated significant clinical efficacy. Five studies^[17–19,21,22]^ assessed microbial clearance rates. Additionally, 5 studies^[17,18,20,21,23]^ evaluated the safety of cefoperazone/sulbactam treatment. Table 2 summarizes the outcome measures reported in the included studies.

**Table 1:** Characteristics of included studies.

Country	Study design	Treatment group drug	Control group drug	Treatment/control group (no. of people)	Treatment/control group (age)	Course of disease (d)	Infection site	Outcome measures	Treatment options
India	Multicenter (17)	Cefoperazone Sulbactam	Ceftazidime	154/152	36.4 ± 15.4/35.7 ± 16.3	NA	Gastroduodenal perforation, intestinal perforation, acute appendicitis, peritonitis	Effective, significant effect, adverse reactions, bacterial clearance rate	8g/d, 5 to 14 d
China	Single center	Cefoperazone Sulbactam	Cefotaxime Sodium	55/54	NA	NA	Acute appendicitis, acute peritonitis, calculus, cholecystitis with biliary tract infection	Effective, significant effect, adverse reactions, bacterial clearance rate	Intravenous administration, 4 g/d
China	Single center	Cefoperazone Sulbactam	Ceftriaxone Sodium	43/43	42.65 ± 3.65/42.71 ± 3.58	5.56 ± 1.22/5.61 ± 1.18	Spontaneous bacterial peritonitis	Effective, significant effect, adverse reactions, bacterial clearance rate	1 time/d for 14 d
China	Single center	Cefoperazone Sulbactam	Ceftriaxone Sodium	90/90/90	37.1 ± 8.9/38.1 ± 8.9/34.6 ± 7.4	13.9 ± 8.1/12.9 ± 8.6/10.7 ± 6.8	Acute bacterial infection of the bile ducts	Effective, significant effect, adverse reactions, bacterial clearance rat	1 time a day for 7 d
China	Single center	Cefoperazone Sulbactam	Cefotetan	40/40	42.9/47.3	NA	Purulent infection of the abdominal cavity	Effective, significant effect, adverse reactions, bacterial clearance rate	1 time every 12 h, the course of treatment is 6 to 14 d
Japan	Multicenter	Cefoperazone Sulbactam	Cefazoloxime	36/31	NA	NA	Purulent infection of the abdominal cavity	Effective, significant effect,bacterial clearance rate	NA
China	Multicenter	Cefoperazone Sulbactam	Ceftriaxone	95/95	48.8 ± 13.4/51.0 ± 15.0	NA	Acute purulent cholangitis	Effective, significant effect, bacterial clearance rate	1 time before the start of surgery and 2 times a day after surgery

NA = not available.

**Table 2:** Data on outcome indicators included in the study.

Study	Year	Study type	Effectiveness rate	Significant effectiveness rate	Microbial clearance rate	Adverse reactions
Chandraet al (2008)	2008	RCT	Treatment group: 125/154 Control group: 108/152	NA	Treatment group: 65/71 Control group: 52/66	Treatment group: 83/154 Control group: 78/152
Geng et al (2012)	2012	RCT	Treatment group: 53/55 Control group: 48/54	Treatment group: 50/55 Control group: 43/54	Treatment group: 45/51 Control group: 34/47	Treatment group: 3/55 Control group: 3/54
He et al (2020)	2020	RCT	Treatment group: 38/43 Control group: 30/43	Treatment group: 35/43 Control group: 25/43	Treatment group: 41/45 Control group: 33/44	NA
Wang et al (2013)	2013	RCT	Treatment group: 85/90 Control group: 79/90	Treatment group: 80/90 Control group：73/90	NA	Treatment group: 6/90 Control group: 7/90
Yang et al (2010)	2010	RCT	Treatment group: 40/40 Control group: 39/40	Treatment group: 39/40 Control group: 38/40	Treatment group: 33/37 Control group: 24/36	Treatment group: 2/40 Control group: 2/40
Yura et al (1985)	1985	RCT	Treatment group: 20/36 Control group: 22/30	Treatment group: 9/36 Control group: 7/30	Treatment group: 14/16 Control group: 9/13	NA
Zhou et al (2004)	2004	RCT	Treatment group: 94/95 Control group: 91/95	NA	NA	Treatment group: 2/95 Control group: 1/95

NA = not available, RCT = randomized controlled trial.

### 3.3. Study methods

**Figure 2.**
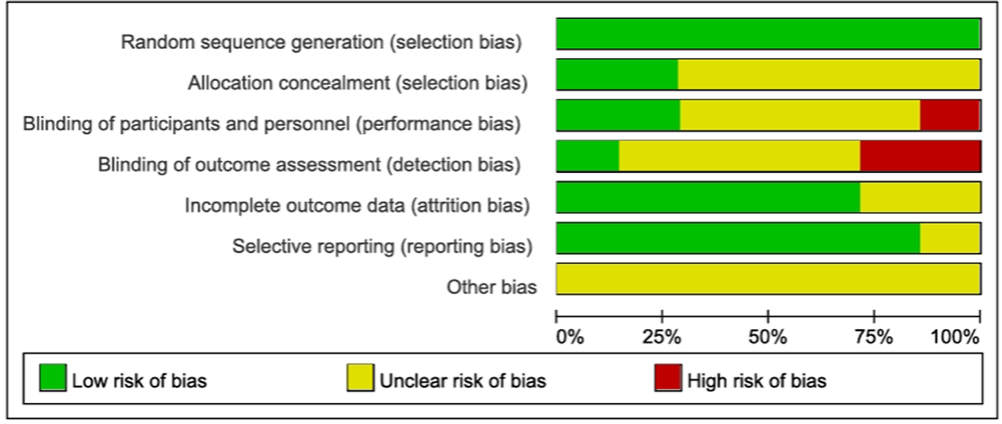
The figure represents the risk of bias assessment for the studies selected in the meta-analysis.

**Figure 3.**
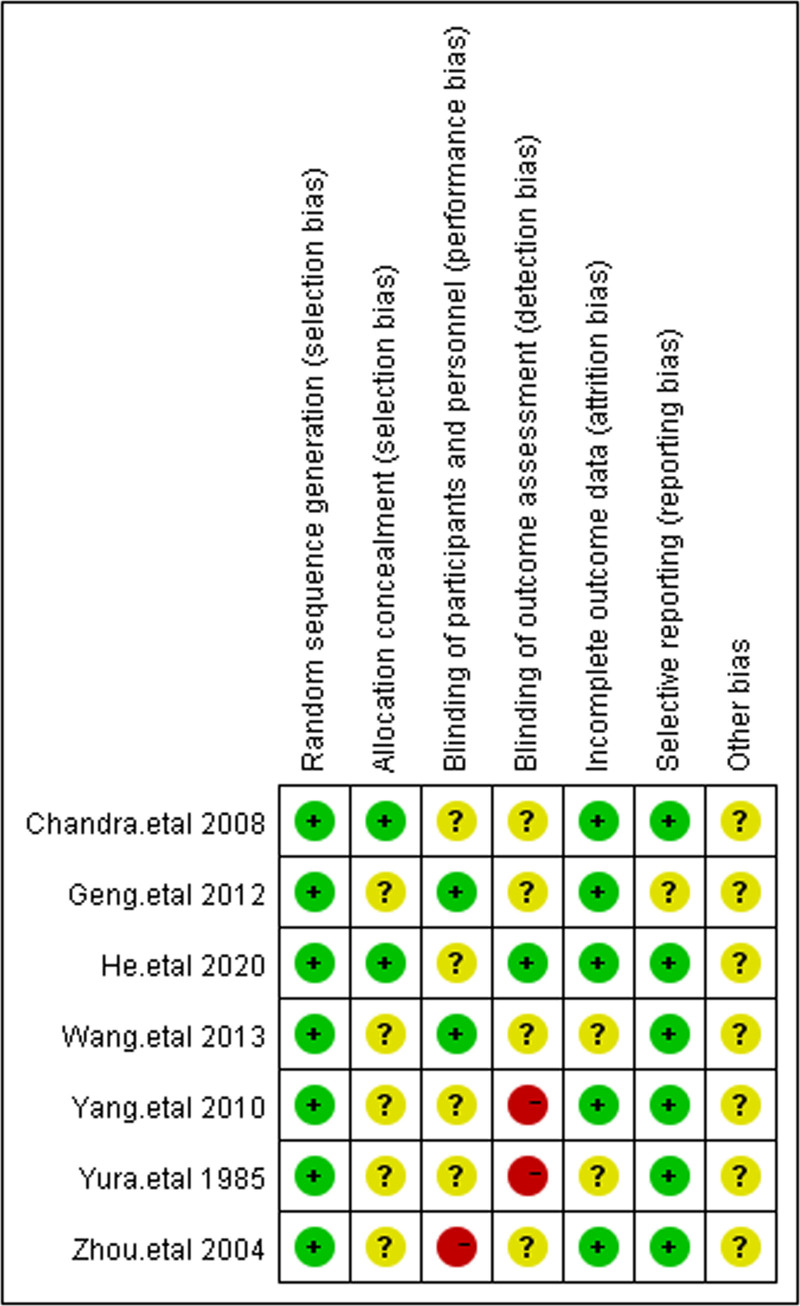
The figure represents the risk of bias assessment for the studies selected in the meta-analysis (2).

The methodological quality assessment results are shown in Figures 2 and 3. All 7 studies reported the use of random allocation methods, with random number tables being used in some, and these were rated as low risk for bias. However, 2 studies^[17,19]^ did not sufficiently describe the allocation concealment process and were rated as having an unclear risk of bias, while other studies were rated as having an unknown risk due to incomplete descriptions. In terms of selective reporting, 6 studies were classified as having a low risk of bias, as they reported all prespecified outcomes. For other potential biases, insufficient data were available to make a judgment across the 7 studies.^[17–23]^

### 3.4. Treatment outcomes

#### 3.4.1. Treatment efficacy

**Figure 4.**
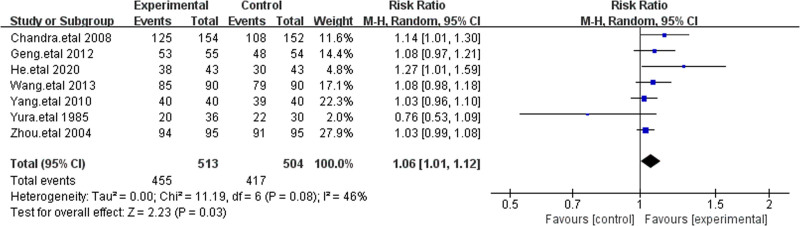
The figure represents a forest plot of the meta-analysis for treatment efficacy.

All 7 studies reported treatment efficacy, including a total of 1017 patients from various countries.^[17–23]^ Among the 513 patients treated with cefoperazone-sulbactam, 455 achieved successful treatment outcomes. In the control group, 417 of the 504 patients experienced successful treatment outcomes. As the heterogeneity among the studies was <50%, we used a fixed-effects model. The treatment success rate for cefoperazone-sulbactam was significantly higher than that of other cephalosporins (RR = 1.08 [1.02–1.13], *P* = .003, Fig. 4). To ensure the robustness of the results, we conducted a sensitivity analysis by sequentially excluding individual studies, and the results remained consistent, indicating that cefoperazone-sulbactam offers superior efficacy in treating IAIs compared to other third-generation cephalosporins (Fig. 4).

#### 3.4.2. Significant treatment efficacy

**Figure 5.**
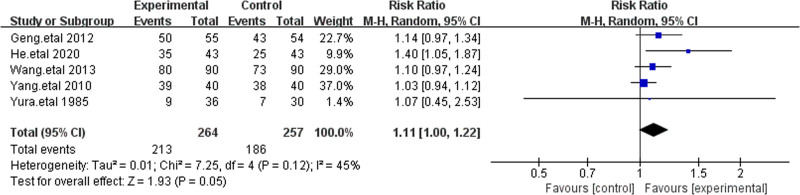
The figure represents a forest plot of the meta-analysis for significant treatment efficacy.

To better reflect clinical practice, we analyzed the significant treatment efficacy of cefoperazone-sulbactam. Patients who were treated with the study antibiotics without relapse or use of additional antibiotics during follow-up were included in this analysis. Five studies reported significant treatment efficacy, involving 264 patients in the treatment group and 257 patients in the control group.^[18–22]^ The results showed that the RR for cefoperazone-sulbactam compared to the control group was 1.13 [1.04, 1.23]. Sensitivity analysis was also performed by sequentially excluding individual studies, and the results remained stable, indicating that cefoperazone-sulbactam offers superior control of IAIs compared to other third-generation cephalosporins (Fig. 5).

#### 3.4.3. Microbial clearance rate

**Figure 6.**
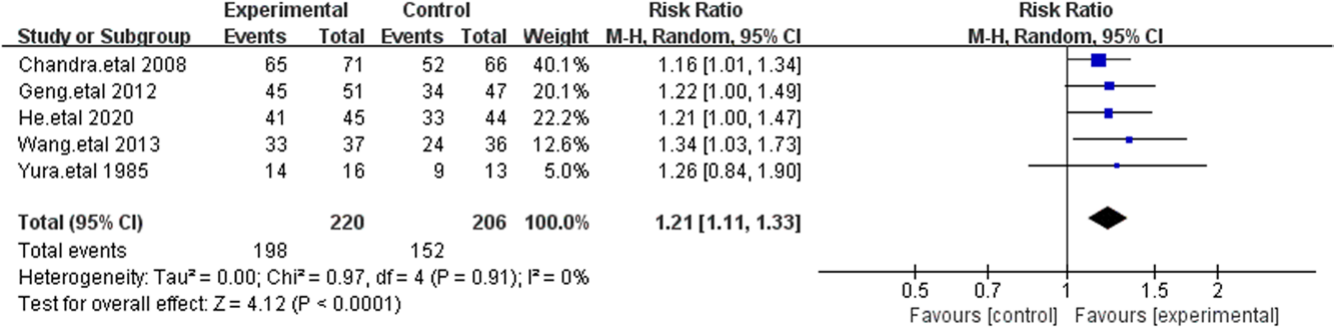
The figure represents a forest plot of the meta-analysis for microbial clearance rate.

Microbial clearance rate refers to the rate at which a specific treatment or natural immune response reduces the number of microorganisms in the body. This measure is often used to assess the effectiveness of antimicrobial drugs or immune responses in eliminating pathogenic microorganisms. We compared the microbial clearance rates between cefoperazone-sulbactam and other cephalosporins. Five studies reported microbial clearance rates, showing that patients treated with cefoperazone-sulbactam had a significantly higher clearance rate than those in the control group (RR = 1.22 [1.11, 1.34]). Sensitivity analysis confirmed the stability of these results, indicating that cefoperazone-sulbactam had a superior microbial clearance rate compared to other third-generation cephalosporins (Fig. 6).^[17–19,21,22]^

#### 3.4.4. Adverse reactions

**Figure 7.**
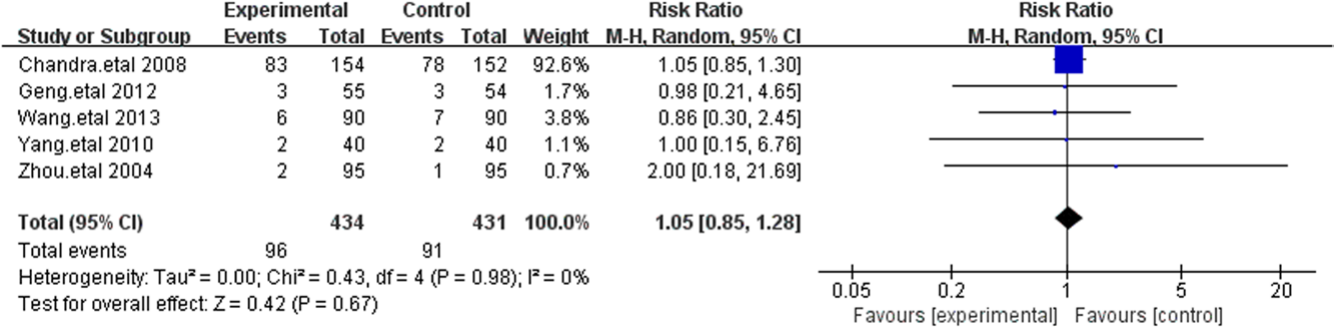
The figure represents a forest plot of the meta-analysis for adverse reactions.

In this meta-analysis, 5 studies reported adverse reactions, involving a total of 865 patients.^[17,18,20,21,23]^ Among patients treated with cefoperazone-sulbactam, rash was the most common adverse reaction, followed by nausea/vomiting. The overall incidence of adverse reactions was 22% in the treatment group and 21% in the control group, with an RR of 1.04 (*P* = .98; *I*² = 0%). All studies that reported adverse reactions indicated no significant differences between cefoperazone-sulbactam and other cephalosporins in terms of safety, demonstrating that cefoperazone-sulbactam is well tolerated (Fig. 7).

#### 3.4.5. Subgroup analysis

**Figure 8.**
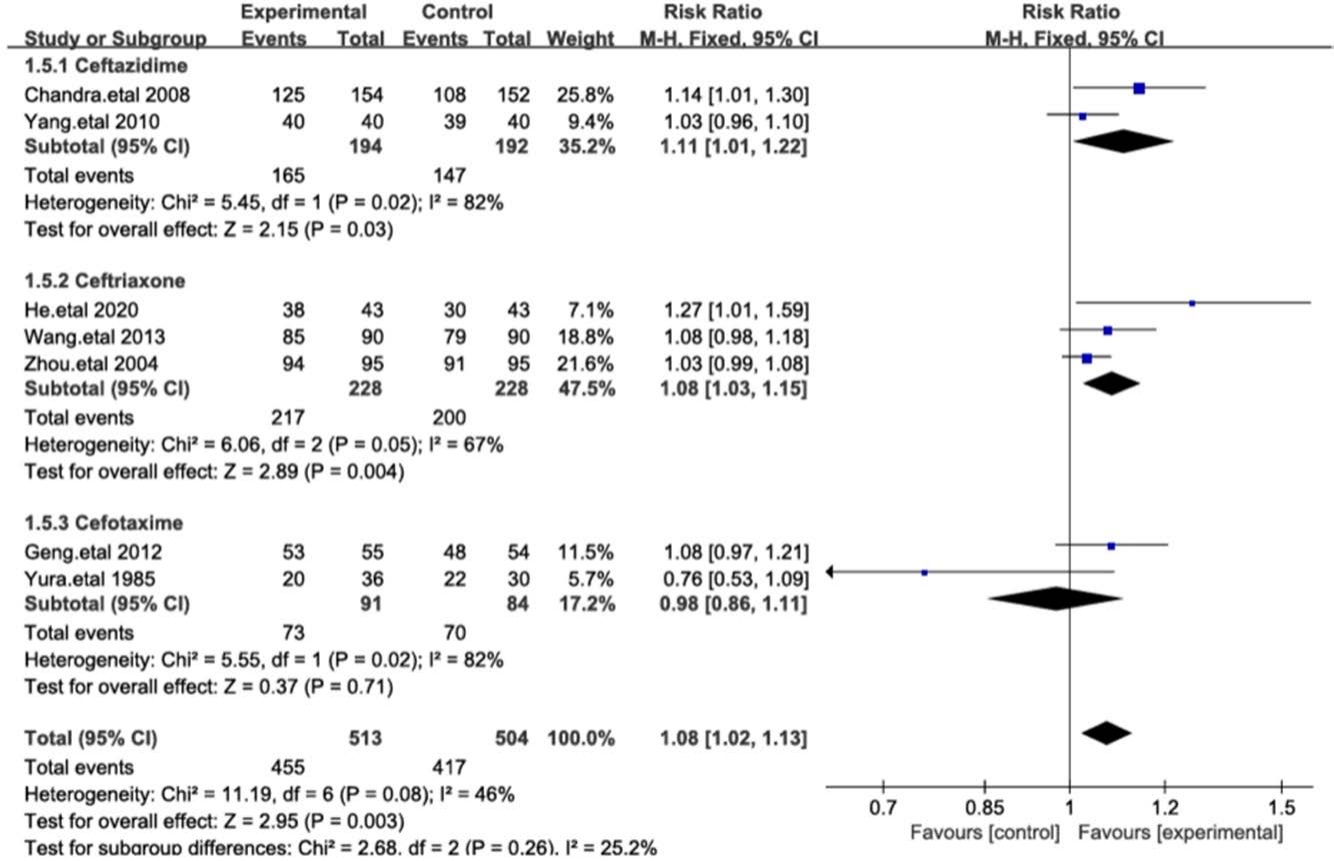
The figure represents a forest plot of the meta-analysis for types of cephalosporins.

**Figure 9.**
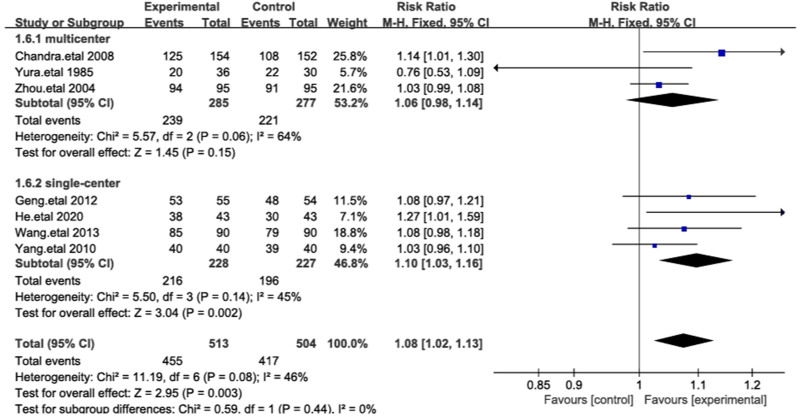
The figure represents a forest plot of the meta-analysis for study design.

To further explore the comparative efficacy of different cephalosporins and cefoperazone-sulbactam, we conducted a subgroup analysis based on the types of cephalosporins used in the control group. Ceftriaxone, ceftazidime, and cefotaxime are commonly used third-generation cephalosporins that exert bactericidal effects by inhibiting bacterial cell wall synthesis. The results showed statistically significant differences between cefoperazone-sulbactam and ceftriaxone, ceftazidime (*P* < .00001), with low heterogeneity among the 3 groups (*I*² = 25%; Fig. 8). We also compared the impact of study design on outcomes, showing that in multicenter studies, despite the lack of statistical significance, cefoperazone-sulbactam exhibited a trend toward superior efficacy compared to other cephalosporins (RR = 1.06). In single-center studies, the comparative results showed significant statistical differences (Fig. 9).

## 4. Discussion

Cefoperazone-sulbactam is a broad-spectrum antibiotic with strong in vitro activity against common pathogens, including Gram-positive, Gram-negative, and anaerobic bacteria. Moreover, the combination of sulbactam with cefoperazone significantly enhances their activity against multidrug-resistant organisms (MDROs), such as extended-spectrum beta-lactamase (ESBL)-producing *Escherichia coli* and *Klebsiella pneumoniae*, as well as carbapenem-resistant *Acinetobacter baumannii*.^[12,13]^In our study, we systematically compared cefoperazone-sulbactam with other cephalosporins, and to our knowledge, this is the first comparison of the efficacy of cefoperazone-sulbactam with other cephalosporins.^[15,24]^

In terms of treatment efficacy, the results indicate that cefoperazone-sulbactam demonstrates superior treatment outcomes compared to ceftazidime, ceftriaxone, and cefotaxime. This treatment advantage remained significant even after sensitivity analyses, including the exclusion of individual studies. Furthermore, the significant clinical efficacy also supports these findings.^[25]^ Additionally, the microbial clearance rate results show that cefoperazone-sulbactam exerts a more effective antimicrobial effect at the infection site in various intra-abdominal infection scenarios. This effect can be explained by the composition of cefoperazone-sulbactam, which combines a third-generation cephalosporin (cefoperazone) with a beta-lactamase inhibitor (sulbactam).^[26,27]^ In complicated IAIs, the primary resistance problem arises from ESBL-producing Enterobacteriaceae. These bacteria are commonly found in hospital-acquired infections and are capable of hydrolyzing and inactivating a wide range of beta-lactam antibiotics, including third-generation cephalosporins, penicillins, and aztreonam. Cefoperazone-sulbactam can inhibit beta-lactamases while effectively eradicating ESBL-producing *E coli* and other Enterobacteriaceae strains that remain sensitive to third-generation cephalosporins.^[28,29]^

Apart from efficacy, safety is another major concern in antimicrobial therapy. Our study demonstrates that cefoperazone-sulbactam is well-tolerated by patients. In terms of adverse reactions, cefoperazone-sulbactam exhibited similar side effects to other cephalosporins, such as allergic reactions. Cephalosporins themselves do not cause allergic reactions; however, during the production, storage, or use of cephalosporins, they can bind or aggregate with large molecular substances like proteins or peptides, forming complete antigens that induce the production of IgE antibodies in the body, leading to rapid allergic reactions. This factor is based on the entire beta-lactam class, and the differences between class-specific side chains and the R1 and R2 side chains provide antigen specificity. For patients with allergic reactions, skin tests have high diagnostic value and can often prevent severe allergic responses caused by drug administration. In summary, cefoperazone-sulbactam has excellent safety in clinical use.

However, this study has some limitations. First, this is a meta-analysis, and despite statistical adjustments for potential confounding factors, the possibility of residual bias cannot be completely ruled out. Second, although the clinical outcomes required for drug therapy were reported across different studies, some studies did not perfectly standardize the evaluation of these outcomes. Additionally, the included studies were primarily single-center trials, and more standardized multicenter studies are needed.^[30–32]^ However, the consistency across different outcome measures, such as cure rates and microbial clearance rates, as well as the consistent comparative results across different drugs, supports the robustness of our findings. We also call for more comprehensive multicenter RCTs on cefoperazone-sulbactam in the future to fully explore its potential for infection treatment from different perspectives.

## 5. Conclusion

Cefoperazone-sulbactam shows superior efficacy compared to other cephalosporins in the treatment of infections, particularly in cases involving multidrug-resistant organisms. It also exhibits a comparable safety profile, making it a valuable option in clinical practice. However, further multicenter RCTs are needed to fully assess its potential in broader clinical applications.

## Author contributions

**Conceptualization:** Jieying Zhang, Li Ju.

**Data curation:** Wenwei Sun, Jieying Zhang, Ke Miao, Li Ju.

**Formal analysis:** Li Ju.

**Investigation:** Wenwei Sun, Jieying Zhang, Ke Miao.

**Methodology:** Wenwei Sun, Ke Miao.

**Supervision:** Yuan Hengjie.

**Validation:** Li Ju, Yuan Hengjie.

**Writing – original draft:** Wenwei Sun, Jieying Zhang, Ke Miao, Li Ju, Yuan Hengjie.

**Writing – review & editing:** Wenwei Sun, Jieying Zhang, Ke Miao, Li Ju, Yuan Hengjie.
